# Handlungsempfehlung zeitlich begrenzter Therapieversuch in der Neurointensivmedizin

**DOI:** 10.1007/s00101-025-01516-7

**Published:** 2025-03-17

**Authors:** Angelika Alonso, Annette Rogge, Patrick Schramm, Urs Münch, Susanne Jöbges

**Affiliations:** 1 Neurologische Klinik, Medizinische Fakultät Mannheim der Universität Heidelberg, Mannheim, Deutschland; 2 Nordseeklinik Helgoland, Helgoland, Deutschland; 3 DRK Kliniken Berlin, Berlin, Deutschland; 4 Klinik für Anästhesiologie und Intensivmedizin (CVK/CCM), Charité – Universitätsmedizin Berlin Charité - Universitätsmedizin Berlin, corporate member der Freien Universität Berlin und der Humboldt-Universität zu Berlin, Augustenburger Platz 1, 13353 Berlin, Deutschland; 5 DIVI-Sektion Ethik, Deutsche Interdisziplinäre Vereinigung für Intensiv- und Notfallmedizin (DIVI) e. V., Schumannstr. 2, 10117 Berlin, Deutschland; 6 DIVI-Sektion Studien und Standards in der Neuromedizin, Deutsche Interdisziplinäre Vereinigung für Intensiv- und Notfallmedizin (DIVI) e. V., Schumannstr. 2, 10117 Berlin, Deutschland; 7 DIVI-Sektion Bewusstseinsstörungen und Koma, Deutsche Interdisziplinäre Vereinigung für Intensiv- und Notfallmedizin (DIVI) e. V., Schumannstr. 2, 10117 Berlin, Deutschland; 8 Klinik und Poliklinik für Neurologie , Universitätsklinikum Carl Gustav Carus der Technischen Universität Dresden, Dresden, Deutschland

**Keywords:** Funktioneller Status, Lebensqualität, Neurointensivmedizin, Prognose, Zeitlich begrenzter Therapieversuch, Functional status, Neurocritical care, Prognosis, Quality of life, Time-limited trial

## Abstract

Viele akute Hirnerkrankungen gehen mit einer akuten Bewusstseinsstörung einher. In einer Notfallsituation werden in der Regel zuerst lebensrettende Maßnahmen ergriffen und eine Intensivbehandlung eingeleitet. Wenn es in den ersten Tagen keine signifikante Verbesserung mit Wiedererlangung des Bewusstseins gibt, entstehen regelmäßig sehr komplexe Entscheidungssituationen. In der Neurointensivmedizin ist ein zeitlich begrenzter Therapieversuch („time-limited trial“, TLT) ein wichtiges strukturierendes Element in der Behandlungsplanung und -kommunikation, als verbindliche Vereinbarung zwischen dem Behandlungsteam und dem Patienten oder dem gesetzlichen Vertreter über ein Behandlungskonzept für einen definierten Zeitraum. Aufgrund der verlängerten neurologischen Rehabilitationsphase kann die TLT in der Neurointensivmedizin auch Wochen oder Monate dauern. Dies erfordert häufig eine abteilungsübergreifende Kommunikation (Akutversorgung/Rehabilitation/Langzeitpflege), eine Neubewertung und Umsetzung in der Neurointensivmedizin. Die Empfehlungen umfassen die Definition, empirische Belege und Umsetzungsvorschläge für eine TLT für kritisch kranke Patienten in der Neurointensivmedizin.

## Abstract (en)

Many acute brain disorders are associated with acute disorders of consciousness. In an emergency situation, life-saving measures are usually taken first and intensive care is initiated. If there is no significant improvement with recovery of consciousness in the first few days, very complex decision-making situations arise regularly. In neurointensive care, a time-limited therapy trial (TLT) is an important structuring element in treatment planning and communication, as a binding agreement between the treatment team and the patient or legal representative on a treatment concept for a defined period of time. Due to the prolonged neurological rehabilitation phase, the TLT in neurointensive care can also last weeks or months. This often requires interdepartmental communication (acute/rehabilitation/long-term care), re-evaluation and implementation in neurointensive care. The recommendations include the definition, empirical evidence and implementation suggestions for a TLT for critically ill neurointensive care patients.

## 1. Einleitung

Eine Vielzahl von akuten Erkrankungen des Gehirns, wie zerebrale Ischämien, intrakranielle Blutungen, hypoxische Enzephalopathien oder Schädel-Hirn-Traumata (SHT), geht mit einer akuten Störung des Bewusstseins einher. In Einzelfällen kann – bedingt durch den vorausverfügten Patientenwillen, das erhebliche Ausmaß der Schädigung und eine anzunehmend sehr unwahrscheinliche Erholungsfähigkeit – eine Sterbebegleitung bereits in der Akutsituation geboten sein.

In der Notfallsituation werden initial meist lebensrettende Maßnahmen – oft inklusive Intubation und mechanischer Beatmung – ergriffen und eine intensivmedizinische Versorgung begonnen. Wenn dann in den ersten Tagen keine deutliche Besserung mit Wiedererlangung des Bewusstseins eintritt, entstehen regelmäßig sehr komplexe Entscheidungssituationen, in denen eine strukturierte Behandlungsplanung und Kommunikation erfolgen müssen. Als wichtiger Strukturgeber bietet sich insbesondere in der Neurointensivmedizin die Vereinbarung eines zeitlich begrenzten Therapieversuchs („time limited trial“, TLT) an. Dieser bezeichnet eine verbindliche Übereinkunft zwischen dem Behandlungsteam und der Patientin oder dem Patienten bzw. der juristischen Stellvertreterin oder dem juristischen Stellvertreter über ein Behandlungskonzept für einen definierten Zeitraum [1, 2].

Da der zeitliche Rahmen neurologischer Rehabilitation abhängig von zugrunde liegender Erkrankung, Alter und Begleiterkrankungen Wochen bis Monate umfassen kann, bedürfen TLT in der Neurointensivmedizin unter Umständen auch sektorenübergreifender (Akutversorgung/Rehabilitation/Langzeitpflege) Kommunikation, Reevaluation und Umsetzung. Damit unterscheidet sich ein TLT in der Neurointensivmedizin häufig von dem TLT bei anderen intensivmedizinischen Erkrankungen mit nahezu infauster Prognose, in dem Patienten nur noch eine sehr geringe Chance für ein positives Outcome eingeräumt wird.

Die vorliegende Handlungsempfehlung umfasst Definition, Empirie und konkrete Vorschläge zur Umsetzung eines TLT bei *schwer betroffenen* neurointensivmedizinischen Patientinnen und Patienten.

## 2. Diagnostische Unsicherheit

Die Neurointensivmedizin ist im diagnostischen und im prognostischen Prozess im Vergleich zu anderen intensivmedizinischen Disziplinen in besonderer Weise von Unsicherheiten betroffen. Darüber hinaus ist in der Neurointensivmedizin aufgrund der Häufigkeit von Bewusstseinsstörungen meist eine stellvertretende Einwilligung auf Basis des mutmaßlichen Patientenwillens in jedes diagnostische oder therapeutische Angebot notwendig. Dies bringt einen zusätzlichen und ebenso vielschichtigen Unsicherheitsfaktor für die gemeinsame Behandlungsplanung mit sich.

Beim Übergang in chronische Stadien einer Bewusstseinsstörung steht vor der prognostischen Einschätzung zunächst die Herausforderung einer richtigen diagnostischen Zuordnung dieser Bewusstseinsstörung. Die Diagnostik muss hierbei immer multimodal, wiederholt und durch Fachexpertinnen und -experten erfolgen [3, 4], um bei diesen komplexen Krankheitsbildern das Risiko von Fehleinschätzungen zu reduzieren. Die große Gefahr einer Fehleinschätzung, insbesondere im Rahmen von bettseitigen Diagnosen, wurde in verschiedenen Studien deutlich [5–9]. Die Nutzung von validierten Skalen kann Unsicherheiten in der diagnostischen Zuordnung reduzieren, aber nicht eliminieren [10]. Dies ist von besonderer Relevanz, da die diagnostische Einordnung im weiteren Entscheidungsprozess einen bedeutenden Faktor für die prognostische Einschätzung und folglich für die weitere Therapiezielfindung darstellt [11–13].

## 3. Prognostische Unsicherheit in der Neurointensivmedizin

Einer Prognosestellung liegt eine evidenzbasierte und probabilistische Annahme über das zu erwartende Behandlungsergebnis zugrunde. Dabei existieren für die häufigsten neurointensivmedizinisch relevanten Erkrankungen jeweils verschiedene Scores, die in der Kommunikation mit Angehörigen^1^ und juristischen Stellvertretern über das patientenzentrierte Therapieziel entsprechend gewertet und kommuniziert werden müssen [14–16]. Dabei ist immer zu beachten: Personenbezogene Daten dürfen nur mit berechtigten Angehörigen (Vorsorgevollmacht, Betreuung, Schweigepflichtentbindung) geteilt werden.

Für den Schlaganfall kommentieren Gao et al.: „Echte Schlaganfall-Expertise besteht nicht nur darin, die Wissenschaft der evidenzbasierten Modelle zu erlernen, sondern – was noch wichtiger ist – die Kunst zu beherrschen, die verfügbaren Erkenntnisse am Krankenbett anzuwenden“ [17]. Zu berücksichtigen ist hierbei zudem der meist nur moderate Vorhersagewert entsprechender Prädiktionsmodelle [18, 19]. Auch die Zugrundelegung großer Datenmengen mit Anwendung von künstlicher Intelligenz zu Prognoseberechnungen [20, 21] wird die Herausforderung der notwendigen Entscheidungsfindung im individuellen Fall nicht vollständig eliminieren können.

Die Prognosestellung in der Neurointensivmedizin wird immer mit Unsicherheit behaftet [22] bleiben, was auch an Einflussfaktoren aufseiten des Arztes oder der Ärztin liegen kann. Hierzu existieren zahlreiche Studien, die unterschiedliche Einflussfaktoren auf Therapieentscheidungen wie beispielsweise Alter, Haltung und Berufserfahrung oder Fachrichtung der Behandelnden [23] zeigen.

„^1^Angehörige sind alle nahestehende Menschen, die in das soziale Netzwerk der Patientinnen und Patienten eingebunden sind, unabhängig davon, ob eine verwandtschaftliche Beziehung besteht. Neben Familienmitgliedern können dies Verwandte, Freunde und Freundinnen, Nachbarn und Nachbarinnen usw. sein, die den Patienten oder die Patientin pflegen, versorgen, unterstützen oder begleiten und im regelmäßigen Austausch zu ihm oder ihr stehen [24].^“^

## 4. Dimensionen der Lebensqualität

Gerade in Situationen mit schweren neurologischen Schädigungen und einem nicht immer gut vorhersagbaren neurologischen Outcome sollte die zu erwartende Lebensqualität als Grundlage für Entscheidungen über das Wohl des betroffenen Menschen herangezogen werden.

Bei der Vermittlung der neurologischen Prognose ist zu beachten: Das Konzept der „Lebensqualität“ ist individuell und multifaktoriell [25]. Die Bewertung der gesundheitsbezogenen Lebensqualität kann durch eine detaillierte Vorhersage der einzelnen Komponenten der gesundheitsbezogenen Lebensqualität erfolgen. So kann das Konstrukt Lebensqualität in die Bereiche physisches Funktionsniveau, psychologisches Funktionsniveau als Oberbegriff für kognitive und emotionale Funktionen sowie soziales Funktionsniveau untergliedert werden [26]. Die Dimension soziale Integration beinhaltet auch gesundheitsunabhängige Aspekte wie (vorbestehende) familiäre Netzwerke oder Peergroups und finanzielle Ressourcen. Bei der Beratung der Angehörigen sollte versucht werden, möglichst konkret auf die krankheitsspezifischen individuellen Subdimensionen einzugehen. Für das körperliche Funktionsniveau sollten z. B. die Subdimensionen Paresen, Schmerzen, Fatigue, Schlaf und autonome Dysfunktion berücksichtigt werden. Das neuropsychologische Funktionsniveau umfasst z. B. die Themenbereiche Bewusstsein, Sprache/verbale Kommunikation, Gedächtnis/Kognition, Antrieb und Neglect mit Einfluss auf individuelle Einschränkungen der Betroffenen. Hierzu gehören auch Subdimensionen, die das psychische Befinden (z. B. Angst, Depression oder Emotionskontrolle) beeinflussen [27]. Während sich die subjektive Wertigkeit der einzelnen Subdimensionen interindividuell sehr unterscheidet und sich mit dem Lebensalter, der Lebenssituation oder Vorerfahrungen ändern kann [28], besteht gegenüber der Bewertung eines Zustandes mit dauerhafter Bewusstseinsstörung in unserer Gesellschaft eine größere Übereinstimmung: als nicht wünschenswert [29]. Doch auch hier besteht keine Einstimmigkeit, da manche Menschen auch ein Leben ohne erhaltenes Bewusstsein dem Tod vorziehen.

Lebensqualität lässt sich entsprechend nicht allgemeingültig konkret definieren [30]. So gibt es häufig eine Diskrepanz zwischen der Fremdeinschätzung der Lebensqualität von Menschen mit Handicap durch externe Beobachter und Beobachterinnen und den tatsächlichen Lebenszufriedenheitsberichten von Menschen mit Behinderungen selbst („disability paradox“) [31]. Gerade für Menschen mit dauerhafter Bewusstseinsstörung bleibt jedoch unklar, was diese Menschen fühlen und erleben, bzw. ob sie dies als Lebenssituation akzeptieren können. Inwieweit individuelle Ziele in Bezug auf Lebensqualität definiert und erreicht werden können, gilt es, in einem Prozess zu begleiten [32, 33].

## 5. Surrogatentscheidungen

**Abb. 1:**
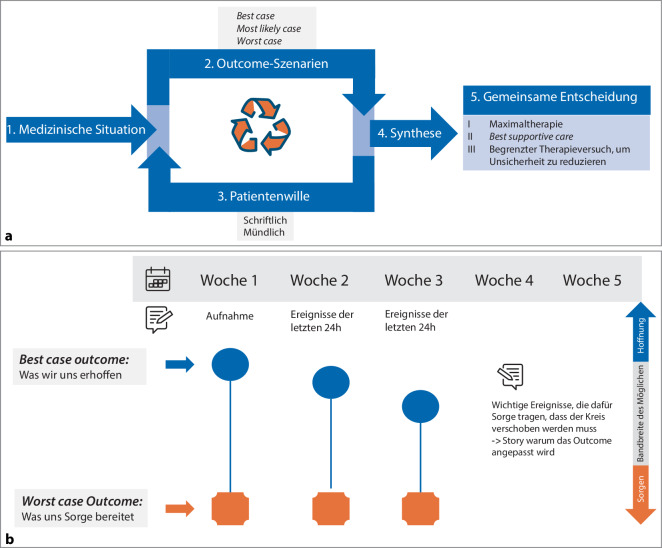
**a** „*Best-case-worst-case“*-Moderationsmodell. (Nach Rogge [39]).** b** „*Best-case-worst-case“*-Grafik – Story line (Visualisierung für Team und Angehörige, nach Wisconsin [37])

Die Beziehungseinheit zwischen Angehörigen und Patientinnen oder Patienten und ihre gemeinsamen Erfahrungen und Wertevorstellungen sind besonders in der Situation schwerer Krankheit von großer Bedeutung. Auf Intensivstationen (ITS) durchleben Angehörige eine emotionale Ausnahmesituation, die von Gefühlen wie Unsicherheit, Überforderung, Angst und dem dringenden Wunsch nach einer bestmöglichen medizinischen Versorgung geprägt ist [34]. In dieser emotionalen Ausnahmesituation müssen Angehörige oft mit Entscheidungen über weitreichende Behandlungsmaßnahmen oder -begrenzungen konfrontiert werden. Diese Entscheidungen umfassen bei Betroffenen mit schweren neurologischen Schädigungen ein Leben mit möglicherweise dramatischen funktionellen und kognitiven Einschränkungen oder Therapiebegrenzungen [35]. In dieser Ausnahmesituation sagen Stellvertreterinnen und Stellvertreter möglicherweise die Behandlungspräferenzen der Patientinnen oder Patienten falsch voraus oder sehen sich nicht in der Lage, Entscheidungen zu treffen [32, 35, 36]. Hier können Entscheidungshilfen wie z. B. „*Best-case-worst-case“-*Szenarien den Angehörigen helfen, die Spannbreite möglicher Behandlungsergebnisse besser zu verstehen, (Infobox 1; Abb. 1a, nach; [39]). Diese Methodik, die ihre Ursprünge in den Wirtschaftswissenschaften hat, wird zum einen zur Darstellung der Entwicklung im zeitlichen Verlauf des Intensivaufenthaltes [37] genutzt, zum anderen dient sie auch als Moderationsmodell in der klinischen Ethikberatung.

### Infobox 1 Das Konzept „best case – worst case“

Das Konzept von „*best case – worst case*“ in Angehörigengesprächen wird oft verwendet, um den möglichen Verlauf einer medizinischen Situation oder eines Behandlungsplans verständlich zu machen.

- *Best case*: Hier wird das bestmögliche Szenario beschrieben, also der optimale Ausgang der Situation. Dies könnte z. B. eine vollständige Genesung oder eine erhebliche Verbesserung des Zustands sein.
- *Worst case*: Hier wird das schlimmstmögliche Szenario skizziert, z. B. eine Verschlechterung des Zustands, dauerhafte Beeinträchtigungen oder der Tod.
- *Most likely case*: Hier wird das Szenario skizziert, das aus Erfahrung bei der Mehrzahl der Patientinnen und Patienten in einer vergleichbaren Situation eintritt.

Das Ziel dieses Konzepts ist es, Angehörigen im Sinne einer „story line“ [35] eine klare Vorstellung von den möglichen Entwicklungen zu geben, um realistische Erwartungen zu setzen und gemeinsam fundierte Entscheidungen zu treffen (Abb. 1b).

Es sollte bedacht werden, dass sich die Einschätzung der Stellvertreterin oder des Stellvertreters bezüglich des Patientenwillens über die Zeit verändern kann. Dies gilt sowohl für eine Fortführung von Behandlungen als auch für Therapiebegrenzungen [35].

Eine besondere Situation stellt sich bei Neugeborenen und Kleinkindern als betroffene Patientinnen und Patienten. Bei diesen kann kein (mutmaßlicher) Wille auf der Grundlage patientenindividueller Wertvorstellungen ermittelt werden, wodurch nur der potenziell sehr unterschiedlich motivierte Wille der gesetzlichen Vertreterinnen und Vertreter Einfluss auf den Prozess nimmt. Diese Situationen stellen für alle Beteiligten eine besondere Herausforderung dar. Sie erfordert bei allen professionell Einbezogenen die Notwendigkeit der Reflexion möglicher eigener Betroffenheit.

## 6. Kommunikation im Team und mit Angehörigen/juristischen Stellvertretern

Sowohl im Team als auch mit Angehörigen/juristischen Stellvertretern ist eine transparente, eindeutige und umfassende Kommunikation [38], die die deutlich längeren Zeiträume der Verläufe in der Neurointensivmedizin berücksichtigt und vermittelt, notwendig.

Die prognostische Unsicherheit und die Etablierung eines TLT brauchen über alle Berufsgruppen hinweg klare Kommunikation hinsichtlich der Therapieziele und Entscheidungsgründe. Das Team sollte dafür sensibilisiert werden, dass im Kontakt mit den Angehörigen möglichst mit einer Stimme gesprochen wird und die patientenindividuell akzeptable Lebensqualität deutlich von dem eigenen Anspruch abweichen kann. Auch in diesem Kontext kann die grafische Darstellung des Szenarios „*best case – worst case“* genutzt werden (Abb. 1b).

In den Gesprächen mit den Angehörigen sollten in regelmäßigen Abständen oder bei akuten Veränderungen mögliche Outcome-Szenarien kommuniziert und mit dem (mutmaßlichen) Patientenwillen abgeglichen werden, um zu einer gemeinsam getragenen Entscheidung zu gelangen [39]. Dabei sollten missverständliche Formulierungen vermieden werden (Tab. 1).

**Tab. 1:** Beispiele für missverständliche Sprache

Der Arzt oder die Ärztin sagt …	Problematisch, weil …	Besser formuliert
*Infaust*	Keine Laiensprache, unkonkret	Ein sehr ungünstiger Krankheitsverlauf bis hin zum Tod
*Stabil*	Suggeriert guten Befund	Außer Lebensgefahr, aber weiterhin sehr kritisch
*Der Test war positiv*	Suggeriert guten Befund	Der Test war auffällig
*Der Patient/die Patientin wird zu 50* *% schwer behindert bleiben*	Wahrscheinlichkeiten beziehen sich immer auf ein Kollektiv und nicht auf ein Individuum	Von 10 Patientinnen und Patienten in der gleichen Situation werden 5 schwer behindert bleiben
*(Nicht) lebenswert*	Suggeriert allgemeingültiges Konzept von Lebensqualität	Entspricht die geschilderte Situation den Wertvorstellungen Ihres/Ihrer Angehörigen?
*Wir können nichts mehr für ihn/sie tun*	Suggeriert, dass der Patient/die Patientin allein gelassen/aufgegeben wird	Wir können sein/ihr Leben nicht retten/verlängern, aber wir können (dafür sorgen, dass er/sie keine Schmerzen hat/nicht leidet/…)
*Wenn es meine Mutter wäre, …*	Suggeriert ein allgemeingültiges und übertragbares Konzept von Lebensqualität	Was ist Ihrem/Ihrer Angehörigen im Leben besonders wichtig?

Hilfreich ist es, die Angehörigen/juristischen Stellvertreter zu fragen, inwieweit sie verstanden haben, mit welchen Einschränkungen der Lebensqualität zu rechnen ist. Dabei sollte auch Emotionen Raum gegeben werden, um diese auffangen zu können [40]. Auch ist abzuklären, inwieweit zusätzliche psychologische, sozialarbeiterische und/oder spirituelle Unterstützung notwendig ist [41]. Feste Ansprechpartnerinnen und Ansprechpartner tragen zur Stärkung des Vertrauens bei. Erleben von Wertschätzung und der Bedeutsamkeit der eigenen Situation ist sowohl für Patientinnen und Patienten als auch für Angehörige essenziell [41, 42].

Können Behandlungsentscheidungen nicht im Konsens getroffen werden, ist die ethische Fallberatung ein Instrument der Wahl.

## 7. Ablauf eines TLT in der Neurointensivmedizin

**Abb. 2:**
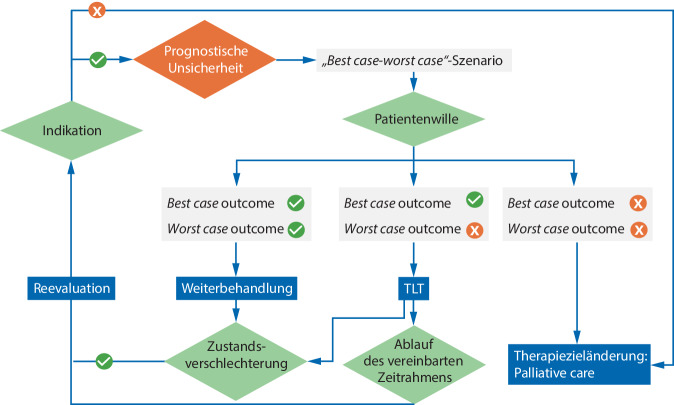
Flowchart zum Umgang mit prognostischer Unsicherheit

Wird ein TLT mit dem Behandlungsteam und der Patientin oder dem Patienten bzw. der gesetzlichen Vertreterin oder dem gesetzlichen Vertreter als Behandlungskonzept für einen definierten Zeitraum vereinbart, so sollte dieser in der Neurointensivmedizin als ein gestuftes Schema angewendet werden (Abb. 2; Infobox 2).

### Infobox 2 Ablauf eines TLT in der Neurointensivmedizin^a^

1. Prognose
  Aktuelle Situation, Behandlungsindikation, Behandlungsoptionen
  Prognose („*best case – worst case“*) durch erfahrenes Behandlungsteam, inkl. Pflegefachpersonen und Therapeutinnen und Therapeuten
2. Ziele + Präferenzen
  Individuelle Patientenziele: Welche Einschränkung ist die Patientin/der Patient bereit zu akzeptieren?
3. Objektive Kriterien
  Individuelle *konkrete *Erfolgs- und Misserfolgskriterien (physisch, psychisch, soziale Dimensionen)
4. Zeitrahmen
  Intensivmedizin
    - Akutphase (bis 72 h)
    - Konsolidierungsphase > 72 h
  Rehabilitationsphase/Postintensivbehandlung
    c. 6–8 Wochen
    d. 6 Monate
5. Mögliche Behandlungsmaßnahmen – Zielvereinbarung
  Festlegung von Behandlungsmaßnahmen + Zeitrahmen
  Festlegung von Therapielimitierung
  Einbindung von Palliativmedizin und Advance Care Planning zu allen Zeitpunkten
6. Kommunikation + Dokumentation mit Information nachbetreuender Einrichtung
7. (Re‑)Evaluation des vereinbarten Therapiezieles zu den definierten Zeitpunkten

^a^ Adaptiert nach Quill/Holloway [2]

### 7.1. Prognose

Zur Erstellung einer Prognose („*best case – worst case*“) werden die aktuelle Situation, die Behandlungsindikation und die Behandlungsoptionen durch ein erfahrenes Behandlungsteam, einschließlich Pflege und Therapeuten, beurteilt. Die Erstellung einer Prognose in der Neurointensivmedizin wird immer mit einer gewissen Unsicherheit behaftet sein [22]. Um in der Situation der prognostischen Unsicherheit dem Patientenwillen im Rahmen einer schweren neurologischen Erkrankung über den Verlauf gerecht zu werden, gilt es zu berücksichtigen, dass Lebensqualität einer patientenindividuellen Einschätzung bedarf und diese sich möglicherweise über die Zeit ändert.

### 7.2. Ziele und Präferenzen

Die Ziele und Präferenzen orientieren sich an den individuellen Behandlungszielen der Patientin, des Patienten und müssen gemeinsam erstellt werden. Welche Einschränkung ist die Patientin/der Patient bereit zu akzeptieren?

### 7.3. Objektive Kriterien

Individuelle Erfolgs- und Misserfolgskriterien sind sehr unterschiedlich und können sich nach Alter, Lebenssituation und Vorerfahrungen verändern. Zu fokussieren ist auf konkrete individuelle Einschränkungen der betroffenen Patientinnen und Patienten wie:

- physische Kriterien wie z. B. Lähmung, Schmerz, Müdigkeit, Schlaf und autonome Dysfunktion,
- psychische Kriterien (wie z. B. emotionale Stabilität, Zufriedenheit) und neuropsychologische Kriterien (z. B. Bereiche wie Bewusstsein, Sprache/verbale Kommunikation, Gedächtnis/Kognition, Antrieb und Neglect),
- soziale Aspekte (z. B. zwischenmenschliche Beziehungen, soziale Anerkennung)

### 7.4. Sinnvolle Zeitrahmen für TLT in Neurointensivmedizin

#### Intensivstation.

In der *Akutphase* kann zunächst ein Zeitraum von 72 h helfen, eine gewisse prognostische Sicherheit über das Ausmaß der eigentlichen zerebralen Schädigung und Überlebenswahrscheinlichkeit zu bekommen [43–45].

Als weiteren Zeitraum für einen TLT in der Neurointensivmedizin bietet sich die Zeit bis zur klinischen *Konsolidierung* der intrakraniellen Folgen (z. B. Ödemrückgang, Blutungsresorption) mit konsekutiver Reduktion der Sedation an. Hier sollte es dann möglich sein, neben den bildgebenden Befunden auch klinisch das reale Schädigungsmuster zu eruieren und in der Prognoseabschätzung mitzuberücksichtigen. Wichtig ist hier auch, die kontextsensitive Halbwertszeit der initial eingesetzten Sedativa zu beachten. Ein TLT ist auch dann zu erwägen, wenn weitere intensivmedizinische Komplikationen auftreten, die ein angestrebtes Behandlungsziel unsicher machen [38].

#### Rehabilitationsphase/Postintensivbehandlung.

Für die Frührehabilitation gibt es diesbezüglich klare Vorgaben durch die Bundesarbeitsgemeinschaft für Rehabilitation (BAR e.V., https://www.bar-frankfurt.de/fileadmin/dateiliste/_publikationen/reha_vereinbarungen/pdfs/GEReha-Prozess.BF01.pdf). Eine neurologische Frührehabilitation dauert in der Regel 6 Monate. Sie kann abgebrochen werden, wenn innerhalb eines 6‑Wochen- bis 8‑Wochen-Fensters keine Fortschritte zu verzeichnen sind. In der OPS wird darüber hinaus klar aufgeführt, dass wöchentliche Stationskonferenzen mit allen an der Rehabilitation beteiligten Mitarbeiterinnen und Mitarbeitern der Rehaklinik durchgeführt werden müssen, und hier müssen die Fortschritte dokumentiert werden und ggf. neue Ziele definiert werden.

In dieser Phase sollte Patientinnen und Patienten und auch den Angehörigen erklärt werden, dass Rehabilitation kein linearer, sondern eher dynamischer Prozess ist und dieser aufgrund von Komplikationen auch Rückschritte beinhalten kann. Zu diesen Zeitpunkten ist eine erneute Reevaluation der Behandlungsoptionen notwendig, die nicht zwangsläufig zu einer Therapiezieländerung führen muss. Ebenso können Kontextfaktoren der Rehabilitation, die pharmakologischen und nichtpharmakologischen Therapien, Co-Morbiditäten und andere Faktoren diese Dynamik beeinflussen.

Wenn es in dieser Zeit zu einer für die Patientin oder den Patienten akzeptablen klinischen Verbesserung der Situation kommt, kann sich ein abschließender deutlich längerer TLT-Zeitraum anbieten. Dieser Zeitrahmen eines TLT muss sich an den erwartbaren Trajektorien der Erholung orientieren. Typische Trajektorien der Neurorehabilitation zeigen zunächst einen steileren Anstieg der Funktionen über die Zeit, gefolgt von einer abflachenden Kurve mit asymptotischer Annäherung an ein Plateau [46], auch wenn verlässliche Zeitangaben in der Literatur nicht verfügbar sind.

Ein erster Evaluationszeitraum von *drei Monaten* erscheint für neurointensivmedizinische Erkrankungen mit akuter Hirnschädigung und initialem Koma wie die hypoxisch-ischämische Enzephalopathie, das schwere SHT oder den schweren hämorrhagischen oder ischämischen Schlaganfall angemessen, wenn eine Änderung der Bewusstseinslage zu mindestens minimaler Interaktionsfähigkeit als Therapieziel definiert wird [47]. Bei persistierendem Koma bzw. Syndrom reaktionsloser Wachheit über mehr als drei Monate ist ein Wiedererlangen des Bewusstseins wenig wahrscheinlich [48, 49]. Insbesondere bei jüngeren Betroffenen mit einem SHT sind jedoch Ausnahmen möglich.

Das Therapieziel hinsichtlich der Erreichbarkeit des langfristig funktionellen Niveaus in den verschiedenen Dimensionen der Lebensqualität sollte nach etwa *sechs Monaten* evaluiert werden. Zu diesem Zeitpunkt ist in der Regel ein asymptotischer Verlauf der Regenerationskurve erreicht, mit zwar weiterem, aber insgesamt deutlich geringerem Erholungspotenzial [50]. So gelingt unter intensiven multimodalen Rehabilitationsmaßnahmen das Überwinden eines Syndroms reaktionsloser Wachheit bei mehr als der Hälfte der Patientinnen und Patienten mit schwerer Hirnschädigung innerhalb der ersten 6 Monate, nach einem bis 2,5 Jahren nur noch bei 1 %. Eine Verbesserung aus einem minimalen Bewusstseinszustand ist innerhalb der ersten sechs Monate die Regel, während nach einem bis 2,5 Jahren nur noch jeder 6. Patient oder Patientin eine Verbesserung zeigt [51].

### 7.5. Mögliche Behandlungsmaßnahmen – Zielvereinbarungen

#### 7.5.1. Realitätsbezogene Behandlungsmaßnahmen - Limitierungen 

Neurologische Erholung ist in der Regel nicht allein durch spezifische intensivmedizinische Maßnahmen zu erreichen, sondern erfordert eine intensive multimodale Rehabilitation über einen prolongierten Zeitraum. Zur Ermöglichung eines Rehabilitationsaufenthalts sind bei Patientinnen und Patienten mit schwerem neurologischem Defizit und/oder anhaltender Bewusstseinsstörung eine längerfristige Sicherstellung der Ernährung, in der Regel über die Anlage einer perkutanen endoskopischen Gastrostomie, sowie ggf. eine Sicherung der Atemwege mittels Tracheotomie notwendig. Die Belastung durch die Eingriffe muss in die Gesamtabwägung miteinfließen. Auch palliativmedizinische Behandlungskonzepte sollten als Behandlungsoption mitgedacht werden [52]. Maßnahmen, die nicht gewünscht sind oder als nicht sinnvoll erachtet werden, müssen definiert und kommuniziert werden. Hierzu zählen insbesondere Therapielimitierungen.

#### 7.5.2. Zielvereinbarungen

In den Zielvereinbarungen müssen konkrete Zielkriterien, die ein realistisches und akzeptables Therapieziel für die Patientin oder den Patienten umfassen, benannt werden. Auch die zum Erreichen des Therapieziels durchgeführten Maßnahmen müssen für die Patientin/den Patienten erträglich sein. Das übergeordnete Therapieziel sollte in der Neurointensivmedizin ein individuelles funktionelles Niveau beschreiben. Es muss überprüfbar sein.

In der akuten Phase auf der Neurointensivstation können als Zielvereinbarung das Überleben und die dafür ggf. notwendigen operativen/interventionellen Maßnahmen definiert werden. Auch ist die Möglichkeit eines irreversiblen Hirnfunktionsausfalls (IHA) zu bedenken und damit eine mögliche Organspende als alternatives Behandlungsziel in Betracht zu ziehen und ggf. zu kommunizieren [53].

Für die Konsolidierungsphase auf der ITS und den prolongierten Verlauf können zur Zielvereinbarung das Ausmaß von Paresen, eine mögliche Aphasie sowie lang anhaltende Vigilanzminderung herangezogen werden. Neben den körperlichen Funktionen sollten auch die Ebenen Aktivitäten und Partizipation (Teilhabe) sowie psychosoziale Faktoren einbezogen werden.

Hierbei kann eine Orientierung an der *International Classification of Functioning, Disability and Health *(ICF) hilfreich sein [54].

Für die Therapiezielfindung sollten möglichst positive Formulierungen des funktionellen Niveaus (Welche Funktion soll möglich sein?) genutzt werden. So ist das Erreichen eines höheren Funktionsniveaus wie z. B. verbale Kommunikation auch für medizinische Laien gut beurteilbar (*Bundesgesundheitsblatt* [55, S. 43 S. 178 ff]).

Eine korrekte Klassifikation von Bewusstseinsstörungen setzt allerdings neurointensivmedizinische Erfahrung voraus und sollte durch entsprechend qualifiziertes Fachpersonal erfolgen [9]. Laborchemische, bildgebende oder andere Surrogatparameter sind in der Prognoseeinschätzung hilfreich, aber als Zielkriterien im Rahmen eines TLT nicht sinnvoll einsetzbar.

### 7.6. (Re)-Evaluation des vereinbarten Therapieziels zu definierten Zeitpunkten

Eine Reevaluation des aktuellen neurologischen Befundes sowie der prognostischen Einschätzung erfolgt im Verlauf zu den im TLT definierten Zeitpunkten. Außerdem ist diese zum Zeitpunkt der Entlassung von der Intensivstation, bei jeder neu auftretenden (auch nichtneurologischen) Komplikation, die die Gesamtprognose beeinflussen kann, und bei neurologischer Zustandsänderung angezeigt.

Mögliche Szenarien im Rahmen der (Re)-Evaluation eines TLT in der Neurointensivmedizin können sein:

- Bei Verbesserung des neurologischen Zustandes erfolgt wie vereinbart eine Weiterbehandlung. Bei Bewusstseinsstörungen ist hier auf subtile Zeichen eines (partiell) erhaltenen Bewusstseins zu achten.
- Bei deutlicher Verschlechterung des neurologischen oder des Gesamtzustandes ist angesichts der kritischen Prognose meist nicht mehr mit einem Erreichen des Therapieziels zu rechnen und eine Therapiezieländerung erforderlich.
- Wurde das Therapieziel bis zum Ende des vereinbarten Zeitraums nicht erreicht, ist ebenfalls eine Therapiezieländerung erforderlich.
- In Ausnahmefällen kann bei deutlicher Verbesserung des neurologischen Funktionsniveaus, das noch nicht dem angestrebten Niveau entspricht, ein zweiter TLT vereinbart werden. Voraussetzung hierfür ist die prognostische Einschätzung, dass das individuelle Therapieziel noch erreichbar ist.

## 8. Zusammenfassung

Die Herausforderungen einer TLT in der Neurointensivmedizin sind vielfältig und gehen über das Konzept des TLT in der nahezu infausten Prognose auf der ITS hinaus.

Ein TLT in der Neurointensivmedizin kann sinnvoll eingesetzt werden, ist jedoch herausfordernd, durch

- die Ungewissheit bei der Bestimmung und Charakterisierung der Langzeitprognose,
- die fehlende Evidenz,
- ein Bias der Behandelnden/Uneinigkeiten im Team,
- die Unklarheit über geeignete patientenzentrierte Endpunkte/Ziele,
- den Einfluss von Stellvertreterentscheidungen und -aussagen mit der Unsicherheit über akzeptable Ergebnisse oder Behinderungsgrade.

Es gilt zu berücksichtigen, dass Lebensqualität einer patientenindividuellen Einschätzung bedarf und diese sich möglicherweise über die Zeit ändert. Zielvereinbarungen mit festgelegten Zeiträumen im Sinne eines (prolongierten) zeitlich begrenzten Therapieversuches können hier die Evaluation unterstützen.

In der neuromedizinischen Ausbildung sollten das Erlernen der Kommunikation über Prognoseunsicherheit, die Anwendung des TLT und die Beurteilung von Langzeitverläufen obligatorisch sein. Der TLT sollte auch in der Neurorehabilitation eine selbstverständliche Option bei prognostischer Unsicherheit sein. In diesem Zusammenhang sollten Rotationen und regelhafte Fallnachbesprechungen und der Austausch zwischen Akutmedizin und Rehabilitationseinrichtungen etabliert werden [56].
